# Resilience in the context of conflict‐related sexual violence and beyond: A “sentient ecology” framework

**DOI:** 10.1111/1468-4446.12931

**Published:** 2022-02-26

**Authors:** Janine Natalya Clark

**Affiliations:** ^1^ Birmingham Law School University of Birmingham, Edgbaston Birmingham UK

**Keywords:** conflict‐related sexual violence, posthumanism, resilience, sentient ecology, social‐ecological systems (SES)

## Abstract

In his research with Indigenous Evenki people living in Arctic Siberia, Anderson introduces the concept of “sentient ecology”, defined as “the mutual interrelation of person and place”. This interdisciplinary article starts from the basic premise that sentient ecology is relevant for research on resilience, and it aims to demonstrate this in two key ways. First, it uses sentient ecology as a novel framework for thinking about resilience, with a particular focus on conflict‐related sexual violence (CSRV)—an area of scholarship that to date has given very little attention to the concept of resilience. The article locates resilience in the fluid and dynamic interactions between individuals and their social ecologies. What sentient ecology contributes in this regard is a different way of thinking about these interactions. In particular, it highlights some of the ways that female and male victims–/survivors of CRSV actively utilize and engage with the more‐than‐human living world around them in the process of rebuilding and moving on with their lives. Second, the article uses sentient ecology as a framework for thinking in new “sentient” ways about social‐ecological systems (SES)—and how the social and ecological parts of these systems communicate with each other. Taking this a step further, it argues that sentient ecology offers a potential basis for developing more posthumanist accounts of resilience as an extension of SES.

## 1 INTRODUCTION

This article begins with a thought; what Morton (2010, p. 7) calls “the ecological thought”, defined as “the thinking of interconnectedness”. However, the ecological thought is more than just a thought that occurs “in the mind”. It is also “a practice and a process of becoming fully aware of how human beings are connected with other beings—animal, vegetable, or mineral” (Morton, 2010, p. 7). At the center of this research is a concept—“sentient ecology”—that both reflects and illustrates the ecological thought and the significance of interconnectedness. In his anthropological research with Indigenous Evenki people living in Arctic Siberia, Anderson (2000, p. 116) defines sentient ecology as “the mutual interrelation of person and place”. This interdisciplinary article does something new and original with the concept, with the aim of demonstrating its wider application and relevance.

First, the article uses sentient ecology as a framework for thinking about resilience, with a particular focus on conflict‐related sexual violence (CSRV). Significantly, extant scholarship on CRSV to date has given little attention to the concept of resilience, for reasons that will be discussed. To be clear from the outset, this article is not making a normative argument that victims−/survivors^1^ of CRSV “ought to be ‘resilient’” (MacKinnon & Derickson, 2013, p. 262), and nor is it putting the onus on individuals to *be* resilient. Rather, it theorizes resilience as a process that is “co‐facilitated by individuals and the systems of which individuals are part” (Theron et al., 2021, p. 361). In short, it adopts a social‐ecological approach that emphasizes “the social and ecological systems and associated resources that are important to human resilience, including supportive relationships, quality education opportunities, meaningful employment, well‐being‐promoting built and natural environments, and enabling cultural heritage” (Theron et al., 2021, p. 361; see also Moletsane & Theron, 2017, p. 3; Ungar, 2012, p. 15).

Sentient ecology offers a different way of thinking about the interactions – and the nature of these interactions – between individuals and their social ecologies.^2^ This is particularly significant for scholarship and policy discussions about CRSV, which have often given little attention to the protective resources that victims−/survivors of such violence may have within their social ecologies (Clark, 2021a). In particular, sentient ecology highlights some of the ways that female and male victims−/survivors of CRSV actively utilize and engage with what they have around them in the process of rebuilding and moving on with their lives. It thus resonates with and speaks to an expanding body of research examining the social and political agency of individuals (and communities) affected by conflict‐related sexual and gender‐based violence (see, for example, Berry, 2018; Kreft, 2019; Oliveira & Baines, 2021; Touquet & Schulz, 2021; Zulver, 2016).

Second, and building on the above, the article utilizes Anderson’s concept of sentient ecology to make a bigger argument about resilience. While there exists a vast corpus of literature exploring resilience from multiple disciplinary perspectives (see, for example, Adger, 2000; Bourbeau & Ryan, 2018; Pascual‐Leone & Batrez‐Faz, 2021; Rutter, 1989; Ungar, 2021), in the last 20 years or so there has been a growing emphasis on interdependent social and ecological systems (SES; see, for example, Berkes et al., 2003; Faulkner et al. 2018; Walker et al., 2004). Colding and Barthel (2019) underline that “SES discourse is a steadily growing knowledge field”. Yet, they also point to a lack of definitional clarity surrounding SES (Colding & Barthel, 2019). Significant in this regard is Schlüter et al.’s (2019) observation that “Interactions between humans and nature have been at the core of SES research for a long time, but doing justice to this interdependence when analyzing SES still remains a challenge”. Responding to this challenge, this article uses sentient ecology as a framework for thinking in new “sentient” ways about SES and “the links and feedback mechanisms” between them (Cretney & Bond, 2017, p. 11). Taking this a step further, it argues that sentient ecology offers a potential basis for developing more posthumanist accounts of resilience, and richer conceptualizations of SES, that do not “fix the boundary between ‘human’ and ‘nonhuman’” (Barad, 2003, p. 821).

The article begins by exploring some of the possible reasons why extant scholarship on CRSV has neglected resilience and argues that the concept of sentient ecology can help to bridge this gap. The second section is methodological and discusses the fieldwork that underpins the article, namely interviews with victims−/survivors of CRSV in Bosnia‐Herzegovina, Colombia and Uganda. The third section empirically demonstrates the significance of sentient ecology in the context of CRSV and illustrates a linkage between sentient ecology and resilience. The conclusion reflects on the broader relevance of sentient ecology for social‐ecological theorizations of resilience and, ultimately, for new posthumanist work on SES.

## 2 THE WIDER CONTEXT

### 2.1 Resilience and CRSV

There is extensive research on the issue of CRSV (see, for example, Baaz & Stern, 2009; Kirby, 2013; Nordås & Cohen, 2021; Revkin & Wood, 2021; Schulz & Touquet, 2020). This scholarship has emphasized, inter alia, the myriad ways that such violence directly affects the lives of victims−/survivors, including physically and psychologically/emotionally (Akinsulure‐Smith, 2014; Dossa et al., 2014; Liebling‐Kalifani et al., 2011; Zalesne, 2020). In contrast, much less attention has been given to how victims−/survivors deal with their experiences and find ways to move forward with their lives (see, however, Coulter, 2009; Laxminarayan & Durr, 2019; Oliveira & Baines, 2021; Schulz & Ngomokwe, 2021). Particularly striking in this regard is the neglect of resilience. While there are some brief references to it, substantive and in‐depth engagement with the concept remains largely lacking—a gap that this author’s own research has sought to address (see, for example, Clark, 2021b, 2021c).

As one illustration, a report by Edström et al. (2016, p. 5), focused on northern Uganda, finds that “despite pervasive discrimination, groups of male survivors [of CRSV] have been able to develop resilience and mutual support through collective action”. However, as the term resilience is left undefined, the report does not sufficiently demonstrate how these men have developed resilience; and the linkage that it appears to make between resilience and the “capacity of male survivors to organize collectively” is not fully explained (Edström et al., 2016, p. 5). Interestingly, Nordås and Cohen (2021, p. 202) argue that “Among sexual violence survivors and their families, studies have found inspiring evidence of resilience and growth”. Yet, they only cite one study (Koos, 2018) in support of this. Zraly et al.’s research (2013), based on 16 months of ethnographic fieldwork in Rwanda, offers a deeper analysis of resilience, albeit one that is specifically focused on the concept’s relationship with motherhood.^3^

The bigger point is that when scholars do refer to resilience in the context of CRSV, they primarily approach it from a psychological or socio‐psychological perspective,^4^ which misses some of the concept’s richness and “multiple origins…within disciplines as varied as psychology, social work, engineering, and ecology” (Bourbeau, 2018, p. 24). As a lens for thinking about resilience, part of the utility of Anderson’s sentient ecology is precisely that it brings some of these disciplines—specifically psychology and ecology—together in a novel way.

It is important to acknowledge that resilience is not an uncontentious concept. One set of critiques associates it with a wider neoliberal agenda that effectively “redistributes responsibilities—and possibilities of blame” from governments to citizens (Dunn Cavelty et al., 2015, p. 7). A “resilient subject”, in short, is one “that must permanently struggle to accommodate itself to the world…a subject that accepts the disastrousness of the world it lives in as a condition of partaking in that world” (Reid, 2013, p. 355; see also Chandler, 2013; Joseph, 2013; Welsh, 2014). Viewed through a neoliberal lens, thus, resilience would mean leaving victims−/survivors of CRSV to “positively adapt to change” (Chandler, 2016, p. 14) and “cope with uncertainty” (Howell & Voronka, 2012, p. 4). This particular framing makes the concept of resilience appear highly discordant and ontologically out of place in a field of scholarship and policy making that puts a strong emphasis on the needs and priorities of victims−/survivors—exemplified by the rhetoric of a “survivor‐centred approach” to CRSV (Clark, 2021a).

However, neoliberal critiques of resilience have themselves met with criticism (see, for example, Bourbeau, 2018; DeVerteuil & Golubchikov, 2016; Schmidt, 2015). Bourbeau (2018, p. 22), for example, maintains that “scholars have been busy documenting neoliberal expressions of resilience without paying much attention to expressions of resilience not dictated by neoliberalism” (see also Corry, 2014, p. 257; Simon & Randalls, 2016, p. 6). Relatedly, Corry (2014, p. 264) makes the important argument—which this article itself seeks to demonstrate—that while critics view ecological notions of resilience “as opening the floodgates for a neo‐liberal commoditization of nature and the biosphere”, the inter‐linking of social and ecological systems “could equally make possible a challenge to neo‐liberalism”.

Concurring with Simon and Randalls (2016, p. 7) that “[r]esilience is not ontologically given”, this research theorizes resilience as a deeply relational concept (Luthar, 2006, p. 780) and locates it in the fluid and dynamic interactions between individuals and their wider social ecologies (Ungar & Liebenberg, 2011, p. 127). It defines resilience as “the capacity of both individuals and their environments to interact in ways that optimize developmental processes” (Ungar, 2013, p. 256). What is significant about this definition is that “it purposely decenters individuals to avoid blaming them for not flourishing when there are few opportunities to access resources” (Ungar, 2013, p. 256). Conceptualizing resilience in this way is therefore useful for drawing attention to where these social ecologies are failing or deficient—which directly challenges neoliberal interpretations of resilience.

This research also challenges the argument that resilience forces us “to become active participants in our own de‐politicization” (Evans & Reid, 2015, p. 156; see also Evans & Reid, 2014). In particular, its discussion of resilience is not about depoliticizing CRSV and elevating victim−/survivor coping over international and national efforts to pre‐empt and tackle such violence (Olsson et al., 2020; Pruitt, 2012); and nor is it about instrumentalizing victims−/survivors of CRSV as “implementers” of global resilience policies that they have few opportunities to resist (Bargués‐Pedreny & Martin de Almagro, 2020, p. 343). On the contrary, this article’s approach to resilience highlights the protective factors that victims−/survivors have within their social ecologies (including children, families, land and non‐governmental organizations [NGOs]), and how they use these resources not only in rebuilding their lives but also, inter alia, in advocating for change, giving back to their social ecologies and resisting structural violence and oppression.

As an interdisciplinary piece of work on resilience that recognizes the latter’s “multiple relations with other disciplines, concepts, and approaches” (Bourbeau, 2018, p. 24), the key aim of this article is to explore and demonstrate what Anderson’s concept of sentient ecology can bring to social‐ecological ways of thinking about resilience—and the relevance of this for the study of CRSV. It is important to acknowledge that existing scholarship on resilience, from various disciplines, has widely explored the significance of broad environmental factors. To take just one example, as scholars moved from psychological to more contextual socio‐psychological ways of thinking about children and resilience (see, for example, Masten & Garmezy, 1985; Werner & Smith, 1992), “Subsequent research led to the delineation of three sets of factors implicated in the development of resilience: (1) attributes of the children themselves, (2) aspects of their families, and (3) characteristics of their wider social environments” (Luthar et al., 2000, p. 544). Anderson’s concept of sentient ecology contributes something new as a way of thinking about resilience and the relevance of the environment. In particular, it illuminates “a relational way of being in the world that invites humans into an emergent and reciprocal dance with more‐than‐human lifeworlds” (George & Wiebe, 2020, p. 503). Outside the context of Indigenous scholarship (see, for example, Hatala et al., 2020; Morton et al., 2020; Tobias & Richmond, 2014), these "lifeworlds" have received little attention within resilience scholarship—and even less within extant literature on CRSV.

### 2.2 Sentient ecology

In his work in the Taimyr region of Arctic Siberia, Anderson (2000, p. 13) notes that “What is unique about this region is the Putoran alpine plateau”. These mountains, he explains, “have the ecological effect of pushing the treeline zone unusually far past the Arctic Circle”, which has made the area along the edge of the plateau “a very rich and comfortable place to hunt wildlife and raise domestic reindeer” (Anderson, 2000, p. 13). This is due to the ease of quickly changing ecological zones (Anderson, 2000, p. 14). The relevance of the Putoran plateau in this regard brings forth the concept of “ecological edges”, referring to areas where different ecosystems overlap with each other—meaning that they are often highly rich in resources. As one illustration, “by situating a camp or community on a shoreline, people are able to draw from both aquatic and terrestrial habitats to obtain needed goods” (Turner et al., 2003, p. 440).

Beyond their association with biodiversity, edges are also important in the sense of complexifying relationships. In ecosystems, for example, edges are “liminal spaces” constituting “zones of interaction” (Howitt, 2001, p. 240). Discussing the concept of “cultural edges” (Turner et al., 2003), Mulrennan and Bussière (2018) argue that it may have particular utility in “representing indigenous history as a series of encounters with neighboring and distant indigenous groups, settlers, and the state that involved interactions that were sometimes invited and welcome, but often imposed and resisted”.

Although Anderson does not explicitly frame it as such, sentient ecology also exemplifies the significance of edges as sites of interaction. Sentient ecology is thus a highly relational idea that reflects different dimensions of connectivity (Tibet, 2018, p. 231)—a concept that is also partly about where things meet (Cooney, 2004, p. 326)—and “interactive connectivities that promote diversity, complexity, and relationality” (Rose, 2017, p. 491). More specifically, sentient ecology is about the deep interactions and connectivities between individuals and the worlds that they form part of and inhabit. Anderson (2000, p. 116) notes, for example, that being a *tundrovik*—meaning someone who lives on the tundra—“is not only a relational identity in the sense that it is a category encompassing people of multiple language groups and nationalities. It also implies an even stronger set of solidarities and obligations between people and certain places and animals” (see also Anderson, 2017, p. 137). Further elaborating on this, he underlines that “Evenki hunters act and move on the tundra in such a way that they are conscious that animals and the tundra itself are reacting to them” (Anderson, 2000, p. 116, n. 1).

A core aspect of sentient ecology is what Anderson (2000, p. 117) calls “knowing.” Knowing is not only a resource, in the sense of helping people to get themselves out of difficult situations, but it is also about an individual’s relationship with the land. For example, “A competent performance of one’s knowledge earns a person respect, establishes one’s status, but also entitles one to enter into a relationship with the land as an independent and competent person” (Anderson, 2000, p. 120). Anderson juxtaposes Evenki relationships with the land with the relationships officially prescribed for them during the Soviet period. In his words, “Instead of knowing the tundra, contemporary hunters and herders are expected to map the tundra—or at least to understand and respect how government officials imagine and enclose space” (Anderson, 2000, p. 15). The bigger point, thus, is that sentient relationships necessarily play out within a broader context and are not immune to external developments.

One illustration of this is Raban’s book *Passage to Juneau*. It can be read, in part, as dealing with the thematic of lost sentience through its reflections on the invention of the magnetic compass—and what this meant for man’s relationship with the sea. Without any instruments to guide him, “the primitive navigator knew his local sea in the same unselfconscious way that a farmer knows his fields” (Raban, 1999, p. 94). With the arrival of the magnetic compass, however, man became a mere “functionary” who “no longer needed to intuit the meaning of the waves” (Raban, 1999, p. 95). At the same time, however, seas also tell a story of deep and enduring sentient connectivities that persist across time and space (Cooney, 2004, p. 323; George & Wiebe, 2020, p. 499). This article specifically demonstrates that sentient connectivities tell a bigger story about resilience, CRSV and the “interconnectedness that characterize[s] our world(s)” (Tickner & Querejazu, 2021, p. 392).

Extant research on CRSV, including feminist scholarship, has strongly emphasized, in different ways, the concept of relationality that is central to Anderson’s work on sentient ecology. Illustrating Longino’s (1993, p. 111) argument that “[a] key feminist insight is that we are all in relations of interdependence”, scholars have examined how sexual violence in conflict can affect and potentially harm relationships between direct victims−/survivors and their social ecologies—including their families and communities (Christian et al., 2011, p. 239; Denov & Kahn, 2019, p. 152; Mukamana & Brysiewicz, 2008, p. 382; Schulz, 2018, p. 598). If these social‐ecological relationships make up a fundamental part of individuals’ environments, scholarship on CRSV has also addressed and explored the environment in the broader sense of gendered and patriarchal structures that facilitate and legitimize the use of violence—and in particular violence against women (see, for example, Boesten, 2014; Davies & True, 2015; Kaya, 2020; Kreft, 2020). Additionally, it has examined some of the ways that socio‐cultural environments can inhibit or deter victims−/survivors from reporting or speaking about their experiences (Pham et al., 2020; Schulz, 2018; Traunmüller et al., 2019), as well as the challenges of prosecuting CRSV in environments characterized by impunity or breakdown of the rule of law (Kravetz, 2017; Perissi & Naimer, 2020; Zawati, 2014). The environment in the sense of nature and ecosystems, however, has received little attention. Similarly neglected are the sentient connectivities that some victims−/survivors have with the environment—and which help them in the process of dealing with their experiences and moving forward with their lives. Herein lies the wider relevance and utility of sentient ecology beyond the particular context of Anderson’s own research in Arctic Siberia.

## 3 METHODOLOGY

This article draws on fieldwork undertaken in the context of a five‐year research project about resilience and victims−/survivors of CRSV. Through its focus on three countries—Bosnia‐Herzegovina (BiH), Colombia and Uganda—that exemplify different patterns and uses of CRSV over different time scales, the research is analyzing, inter alia, how contextual and social‐ecological diversity shapes expressions of resilience, and how different connectivities cluster, operate and rupture to tell a broader narrative about resilience. The study uses mixed methods to capture the nuances and complexities of resilience within a comparative multi‐site research design.

This article draws specifically on the qualitative data, consisting of one‐to‐one semi‐structured interviews with 63 female and male victims−/survivors of CRSV. The interviewees were selected from a larger dataset of 449 victims−/survivors of CRSV across the three countries (BiH *n* = 126, Colombia *n* = 171, Uganda *n* = 152), all of whom completed a study questionnaire (for an analysis of the questionnaire data, see Clark et al., 2021).^5^ Two particular factors guided the selection of interviewees from the quantitative dataset. First, the questionnaire included the Adult Resilience Measure (ARM), a 28‐item scale divided into individual, relational and contextual sub‐scales (Resilience Research Centre, 2016). The ARM is designed to measure an individual’s protective resources across these three interconnected levels, with a higher overall ARM score indicating a larger number of protective resources to support resilience. ARM scores were used to divide participants in each country into four quartiles, with the aim of exploring whether and how the range of ARM scores translated into the qualitative data in terms of particular themes and patterns, and interviewees were selected from across the quartiles. Second, it was important that selection choices reflected the demographic diversity within the quartiles (including gender, ethnic and age diversity), to respect the heterogeneity of victim−/survivor profiles in each country. The author and two postdoctoral researchers carried out the interviews. All interviews took place between January and July 2019 and were conducted in the relevant local language/s. With the participants’ informed consent, interviews were recorded using fully encrypted digital voice recorders. Ethics approval was granted by the author’s host institution, the research funder and by relevant authorities in each country (for a discussion of some of the ethics issues raised, see Clark et al., 2021).

The interview guide included questions about interviewees' conflict experiences, their lives today and their sources of support. Interviewees were also asked intersectional questions about how their gender, ethnicity and the place where they were born and/or lived today affected how they deal with challenges and adversities. All interviews were transcribed verbatim and translated into English. The transcripts were uploaded into NVivo and coded. The author developed the codebook, which underwent multiple iterations, over a period of 12 months and used thematic analysis (Braun & Clarke, 2006) to identify broad themes, clustered around a central connectivity core (see Clark, 2021d). Fundamentally, all of the themes—which include *“It is not there anymore”: Broken and ruptured connectivities*, *“With them I get through it”: Supportive and sustaining connectivities* and *“We have to live”: New connectivities*—speak to the multi‐dimensional connectivities between individuals and their social ecologies.

It is important to note that only three of the interviewees—all of them Colombian—explicitly used the terms “resilient” or “resilience”. Participants in all three countries, however, variously spoke about the need to be strong, to “fight”, to get on with life, to let go of the past and to have a focus. More significantly, all of them talked about the different ways that their social ecologies, and specifically particular resources within these ecologies—including their families, children/grandchildren, friends and contacts with local NGOs or women’s associations—were helping them to deal with everything that they had gone through and to rebuild their lives (see, for example, Clark, 2021a). What also emerged from the data in this regard were stories of sentient ecology.

## 4 STORIES OF SENTIENT ECOLOGY

In her book *Do Glaciers Listen?*—a question that itself reflects the idea of sentient ecology—Cruikshank, referring to a conference that she attended in southern Yukon in 1982, recalls the words of a First Nation elder in her eighties; “[h]er advice to ‘listen for different stories’ has stayed with me…” (Cruikshank, 2005, p. 76). In the context of this research and its exploration of resilience, part of the interview (and coding) process involved “listening for different stories” about the relationships—both positive and negative—between participants and their social ecologies. Significant in this regard was the active presence in the interviewees' stories of the natural environment and the more‐than‐human living world with which they interacted—an illustration of how “[w]e relate, know, think, world, and tell stories through and with other stories, worlds, knowledges, thinkings, yearnings” (Haraway, 2016, p. 97).

Several of the Colombian interviewees particularly stood out in this regard. When asked what title she would give her life story, one interviewee answered “The Caterpillar.” She further elaborated:

Why 'The Caterpillar?' Because to become what I am today, I had to be a caterpillar. Just think, the caterpillar goes through so many changes and those changes must hurt. [J]ust simply breaking out of the chrysalis…it hurts, it causes pain. In order to become the beautiful butterfly, it has to go through a painful preparation, right? So, that’s what I had to go through (interview, Colombia, March 5, 2019).

Speaking about pain in relation to her understanding of the term “victim”, another Colombian interviewee stressed that the impossibility of forgetting this pain made it imperative to get on with one’s life. In her words, “…as I’ve said before, a person has to try to…to keep going. They’ve [referring to the perpetrators] trodden you down, but you have to burst into new life, like a flower or like a chrysalis, and try to keep going forwards” (interview, Colombia, March 13, 2019). In these two examples, the interviewees were essentially “thinking with” the world around them (Cruikshank, 2012), drawing attention to important relational connectivities in their lives.

In their own work on Colombia, with a particular focus on environmental peacebuilding, Yoshida and Céspedes‐Báez (2021, p. 24) emphasize that:

Considering women and men as connected to and living actively in their ecosystems helps to enrich understanding of the implications of armed conflict for their lives and for their communities, and to highlight the key role of those implications on environmental sustainability, preservation, enrichment and transmission of knowledge in these areas.

Thinking about these connections, however, also means thinking about ruptured connections. It is important in this regard to first contextualize the concept of rupture in relation to SES. The key point is that because SES are in constant movement and flux, ruptures inevitably do occur (Walker and Salt, 2006, p. 85) and they are not out of the ordinary (although they may not be desirable).^6^ Highlighting this, the back loop (release and reorganization phases) of the adaptive cycle (Gunderson & Holling, 2002) is quintessentially about rupture; it is when things break up and come apart. It entails “the collapse of accumulated connections and the release of bound‐up knowledge and capital” (Holling, 2004). This rupture and breaking apart, in turn, mean that the back loop can offer opportunities for things to be significantly different (Walker and Salt, 2006, p. 82). According to Wakefield (2017, p. 86), for example, “What the back loop suggests to us is that the Anthropocene is now a time to explore, to let go—of foundations for thinking and acting—and open ourselves to the possibilities offered to us here and now”. It should be emphasized that changes resulting from the back loop do not necessarily make things better or worse, although they can be positive or negative (Holling, 2004).^7^

When rupture occurs in the context of individuals' experiences of armed conflict and large‐scale violence, the resultant changes can be deeply disruptive to lives that will never be the same again. Interviewees in BiH and particularly Colombia, for example, frequently spoke about forced displacement. What they had experienced in this regard were not “transitional ruptures”, which, according to Wilson (2014, p. 18), can “lead to a sudden strengthening of community resilience”, but more long‐term ruptured relationships with their homes, land and communities. In some cases, forced displacement had also ruptured their ways of life and relationships with the natural environment, accentuating Mitchell’s (2014, p. 5) argument that war “destroys human lives and habitations, but also animal and plant life, landscapes and cultures, as well as the complex linkages between these phenomena” (see also Clark, 2020).

A Colombian interviewee, as one illustration, strongly emphasized her *campesina* (rural/countrywoman) identity and articulated a deep sense of “knowing the land” (Anderson, 2000, p. 117). She knew how to survive off it and not to go hungry; “I learned so much—how to grow plantains, yuca. I know all that stuff. I learned to make *rellenas* [a type of sausage, similar to black pudding], I know how to make *tamales* [a traditional Colombian dish made from corn dough and wrapped in a plantain leaf]”. However, she was now internally displaced in a city, completely disconnected from the world that she had known. The people were different [“Here, the women are not as nice as in the countryside where I used to live”], and not being able to keep animals made her feel “like being imprisoned”. She thus expressed a deep yearning for a physical and emotional reconnection with the particular environment that she had grown up in and which had always been such an important part of her life. In her words:

The only thing I long for, in order to be able to live well, in peace, is to get some farmland and be able to keep cows, pigs, chickens. To have what I long for. That would be the only thing that would enable me to rebuild; for everything to be as it was before in T [name of her village]. It would be having a farm again, being able to keep animals and all the things I had there (interview, Colombia, April 3, 2019).

As a very different example of rupture, some of the Ugandan interviewees spoke about physical injuries and chronic health issues that had affected their "knowing" relationship with the land, in the sense of altering how they interacted with it and sustained themselves and their families. A male interviewee talked about his abduction by the Lord’s Resistance Army (LRA) and the time he spent in the bush. He had been made to carry heavy loads over long distances and this had significantly affected his physical health. In his words: “because of carrying heavy loads, it weakened me, taking away my strength, so that I cannot dig” (interview, Uganda, March 26, 2019). In some cases, moreover, additional factors, such as drought (see Branch, 2018, p. 313), had further contributed to ruptures. A female Ugandan interviewee who suffered sexual violence after being forcibly recruited into the LRA and was now living with HIV explained:

The challenges I face in life are too much drought these days. We do farming, but there would still be no food. How to get it is even harder as my body is still not light [healthy]. It makes getting it [food] hard because I do not have the energy to do casual labour (interview, Uganda, February 19, 2019).

All of these examples, and particularly the second two, in different ways illustrate Cooke et al.'s (2016, p. 832) argument that “human–environment (dis)connection can…be framed as an ‘embodied’ relationship”, reflecting the fact that "humans are not just mentally but also materially and physically immersed in their immediate environments". Experiences of displacement and ongoing health issues had created human‐environment disconnection (and rupture) in the sense that although the interviewees were of course still living in and connected to an environment, they could not materially and physically immerse themselves in it—or parts of it—in the same way that they had previously.

It is important to stress that broken and ruptured connectivities are not necessarily incompatible with resilience. This does not mean, however, that ruptures are productive. Arguably they can be, in some circumstances, but the very fact that they are inherently contextual makes it impossible to make broad claims about what ruptures might lead to. The bigger point is that the underpinning research on which this article draws is seeking to tell a story (or rather multiple stories) about resilience through connectivity, and ruptured connectivities were only one part of interviewees' stories. Demonstrating that human‐environment relationships are heterogenous and dynamic (Cooke et al., 2016, p. 835), some interviewees were actively building new connectivities in their lives, such as creating their own associations (this was particularly the case in Colombia) to help other victims−/survivors of CRSV. In all cases, moreover, broken and ruptured connectivites existed alongside (enduring) supportive and sustaining connecivities in the interviewees' lives. For some of them, their relationships with nature and the more‐than‐human living world around them—which this article frames as examples of sentient ecology—were one of the many connectivities and “affective solidarities” (Tonkiss, 2003, p. 299) that were helping them, in various ways, to deal with their experiences of violence.

To examine this in more detail, the remainder of this section centers on three particular research participants—one in each country—whose interviews were especially rich in examples of sentient ecology. Focusing on just three interviewees allows for a fuller discussion of sentient ecology, what it looks like in different socio‐cultural contexts and its significance for understanding some of the ways that victims−/survivors of CRSV deal with their experiences by drawing on relationships and relational connectivities that extant scholarship has not addressed.

### 4.1 Šefik’s story

“Šefik”,^8^ a Bosniak (Bosnian Muslim), was one of the five men interviewed in BiH. Early in the Bosnian war (1992–1995), he was taken to a camp where he suffered multiple abuses and human rights violations, including sexual violence. He spent a total of 15 months detained in several different camps and emphasized that he had “survived Golgotha”. When asked to give three words to describe himself, he did not answer the question. Instead, he expressed his ongoing struggles (accentuated by his staccato speech), more than 20 years on, to comprehend and make sense of what had happened to him as a man. He told the author:

No…I cannot understand, well…Err…I mean, I am not…Those…I, I, I don’t know, I cannot come to terms with this, that a man {does it} with another man, and forcefully. And among ourselves [referring to the fact that men detained in one of the camps were forced to perform sexual acts on each other], like…And many other things…And now, that someone makes you do this, to have to do this. I cannot understand these people [referring to the camp guards] (interview, BiH, April 10, 2019).

Šefik had not talked to his wife about what he went through in the camps; he felt ashamed and embarrassed. He also briefly mentioned that his experiences, “especially at the beginning, while they were still fresh”, had affected his intimate relationship with her. It was nevertheless striking that this interviewee—in contrast to many of the Bosnian interviewees—overall spoke very little about his family, although he did underscore that “If it was not for my family, who knows what would have become of me”. In contrast, a particularly strong theme in Šefik’s interview—and an illustration of sentient ecology—was the importance of a local lake.

Sometimes he sat with friends by the lake, but mostly he preferred being there alone. It was a place where he went to think and a place where he went to forget. In his words:

Like this, I go down to the lake and, as they say, I think of nothing, like…I go to forget. Or, well, also, I sit here and I love being alone sometimes. Sometimes…Sometimes I just don’t like having anyone here. But there are friends, they come along, and sometimes, again…I stay here alone until midnight and when they all leave, then I think and…That’s it.

When asked the question “Who or what is the source of support in your life?”, Šefik talked about the lake. “This is something that keeps me going here”, he maintained. The lake, however, was not simply a resource in his life. It was something much deeper; something that he was fundamentally connected to—and which was fundamentally a part of him. As he explained: “And, well, this birthplace, everything I have learned here. In fact, the water, water is to me…I possibly would not have returned here ever, but it is my birthplace and this lake that I have had since I was a child. As they say, I was born in the lake”. The lake was a key part of his profound attachment to his birthplace (to which he spoke about being “rooted”, "connected" and “tied”), illustrating the “mutual interrelation of person and place” that constitutes sentient ecology (Anderson, 2000, p. 116). His use of the word "learned", moreover, highlights the “knowing” that Anderson (2000, p. 117) writes about. In short, through Šefik’s “knowing the lake”, he had found a way to live with the past and to get on with life. As he summarized it, “I rest here. I rest mentally. Like this, I observe the ducks, fish. Pigeons come along…Like that, and I do not think about problems. I put the music on, over there, like that…This is it. My life, here”.

### 4.2 Isabella’s story

“Isabella” is an Indigenous woman in Colombia. She had previously lived in an area where three armed groups were active—the Revolutionary Armed Forces of Colombia (FARC), the National Liberation Army (ELN) and the paramilitaries—and was unsure which group the two men who had raped her were linked with. When asked about the impact of this particular experience on her life, Isabella reflected: “For me it’s become something good—not because of what was done to me but rather because of what I’m doing with it now” (interview, Colombia, March 6, 2019). In 2016, she set up her own local association to help other women and girls who suffered violence during the armed conflict. Her work—which was a prominent leitmotif within her interview—was challenging, but it had re‐energized her. In her words, “My life is very busy at the moment. I’m not so passive as I was before”. She also viewed the members of her association as her main source of support, describing them as “my friends who are always around and always ready to see what we need to do, where we are going next, what we are doing and aiming for”.

In contrast to Šefik, Isabella did not express any sense of attachment to a particular place, perhaps due to her ongoing internal displacement. The idea of sentient ecology was more implicit in her interview. If, as Masterson et al. (2017) argue, “…there has been insufficient attention to understanding well‐being as co‐created by people and ecosystems”, the important point is that both interviews, in different ways, provide illustrations of this co‐created wellbeing. Particularly interesting in Isabella’s case, as an example of how the relationship between people and nature “is also an intimate, physical and bodily relationship” (Cooke et al., 2016, p. 836), is the fact that she talked about re‐connecting with her own body—and helping the women in her association to do the same. As she explained, “we are trying to get back in touch with our own bodies…We’re remembering how to feel this body that has always been ours and that we started to lose because of things that happened to us and we could not process. That’s what we are doing—it’s a beautiful thing”. If this process was a significant part of her personal journey, so too was the strong sense of sentient connectedness to the more‐than‐human living world that she conveyed through particular words and images. When asked what title she would give her life story, for example, she answered “My New Dawn”. Elaborating on this, she explained:

You are still just yourself, from the same roots, but stronger and with more support—because support is important; all the little bits of help you get every day are just so important for all the people who make up our daily life. It might be some little creature that keeps you company—hearing the sound of a bird singing in the morning. It’s a little bit of companionship and it’s the everyday things that keep you wanting to live each moment.

Like Šefik, Isabella made several references to water. Moreover, she indirectly attributed sentience to it. It did not “listen” like the glaciers discussed in Cruikshank’s (2005) aforementioned work, but it could communicate through the sounds that it made. In Isabella’s own words: “You sit on the banks of a river and listen to the sound of the water—the water speaks to you, it sings, it murmurs and you just want to keep going back to listen to those murmurs, all that. Music. It’s a rebirth. A new dawn”. She thus expressed “knowing” in the sense of knowing how to listen to the sounds around her—and how to emotionally incorporate them into her own life as part of rebuilding it. More broadly, the "knowing" that she exhibited vis‐à‐vis her sentient ecology extended to her knowledge of how to support others in difficult situations. Isabella talked about her efforts to find her daughter, who was captured and held by paramilitaries for two years, and it was during this time that she had started helping other Indigenous girls. She talked about walking miles with them and then taking them to the banks of a local river to get them to safety. Describing these journeys as “true Odysseys”, she had essentially harnessed “knowing” for the benefit of both her own and the girls’ wellbeing.

### 4.3 Grace’s story

“Grace” is an Acholi woman in northern Uganda. Born in 1984, which made her considerably younger than both Šefik and Isabella, like many of the Ugandan interviewees she gave a detailed account of her war experiences. Soldiers from Joseph Kony’s LRA, which was active in northern Uganda from the mid‐1980s until the mid‐2000s, abducted her when she was 12 years old. She maintained that because of her young age, she had not suffered any sexual abuse while in captivity. It was a government soldier from the Ugandan People’s Defense Force, she explained, who had abused her (following her release from the LRA) “in the way of sleeping” (a euphemism for rape). Talking about the sexual violence, she emphasized: “It touched my life because it is something that was against one’s will…So you find that it burns your stomach [makes you angry]” (interview, Uganda, May 29, 2019).

Grace had faced verbal abuse from members of the community, including her younger sister (an example of a ruptured/broken connectivity with an important part of her social ecology). Describing her life today, however, she opined that there was some positive change; “The bleeding of the heart [sadness/hurt] that used to happen in my life is no longer there. At least my heart is gradually untying itself [loosening].” One of the factors that had contributed to this "untying" was finding out that she did not have HIV; her body remained “light” (healthy) and she had not made her children sick. Her main priority now was finding the money to pay for their schooling, and her relationship with the land was very significant in this regard.

Like many of the Ugandan interviewees, Grace was a subsistence farmer. Her father had initially given her some land, and she and her husband’s knowledge and efforts in cultivating it for two years had allowed them to make enough money to purchase their own land. Grace talked about growing maize, eggplant and soybean. She also planted sesame. The previous year, the beans were ruined and she had only managed to harvest one bag. In contrast, she had succeeded in harvesting larger quantities of sesame; “Then it happened that sesame was selling at a better price, and so I sold each kilogram for 1500 [Ugandan] shillings [approximately £0.30]. I sold one bag and it was able to raise for me enough money to pay for the children’s school”. The combined sale of maize and sesame had allowed her to send all four children to school. She additionally talked about using her two oxen to cultivate other people’s land, which provided a further source of income. This “props my back [supports me]”, she explained.

Kandel (2016, p. 275) underlines that “In rural sub‐Saharan Africa, the value of land cannot be overstated. It forms the basis for livelihoods that include (and combine) agriculture, pastoralism, fishing, and hunting and gathering”. While this significance came through in many of the interviews, so too—as Grace’s interview exemplifies—did the “practical manner in which knowing is performed” (Anderson, 2000, p. 118), and the connectivity between people and land as an expression of sentient ecology. This relationship, in turn, was helping Grace to rebuild and move on with her life. Dwelling on the past, she maintained, served no purpose; “it is said that too much thinking can bring illness to the body. Worries can sometimes bring death to your body”. It was therefore necessary to “let go” of the past, and her performativity of "knowing"—which required a healthy body—was a way of doing that.

## 5 CONCLUSION AND WIDER IMPLICATIONS

In 2016, the signing of a peace agreement between Colombia’s government and the FARC guerrillas officially ended more than 50 years of armed conflict in the country. One of the outcomes of this peace agreement was the establishment of the Special Jurisdiction for Peace (JEP), as the judicial element of a Comprehensive System of Truth, Justice, Reparation and Non‐Repetition. To date, the JEP has issued five resolutions in which it recognizes territory as a victim of Colombia’s armed conflict (Huneeus & Rueda Sáiz, 2021). These are highly innovative developments that recognize “human–environment connections” (Cooke et al., 2016, p. 832) and, thus, embrace a deeper conceptualization of harm that extends to “the disruption of socio‐ecological relations, such as forms of subsistence farming and other cultural practices, as well as disruption of the spiritual world” (Huneeus & Rueda Sáiz, 2021, p. 211).

If war and armed conflict can substantially disturb these relations, this article has shown that rupture is not the entire story. Utilizing the idea of sentient ecology—which Anderson developed in the context of his research with Evenki hunters in Arctic Siberia—as a framework for exploring the stories of victims−/survivors of CRSV in BiH, Colombia and Uganda, it has analyzed the concept as a social‐ecological relationship that was supporting the interviewees in dealing with their experiences and getting on with their lives. In so doing, this research has posited a linkage between sentient ecology and resilience.

While not actually connecting the two concepts, some scholars have nevertheless discussed sentience in relation to resilience—and in particular urban resilience (see, for example, Deal et al. 2017, p. 30, 41; Hollnagel, 2014, p. 224, 225). Going a step further, this article posits that the concept of sentient ecology provides a novel framework for thinking about the relationship between the social and the ecological within SES—and how they communicate with each other. Within existing scholarship, SES are theorized as complex adaptive systems (Folke et al., 2005, p. 443) that “involve great numbers of parts undergoing a kaleidoscopic array of simultaneous interactions…” (Holland, 1992, p. 19). Exploring the sentient dimensions of these interactions shifts the emphasis away from broad system behavior—which by itself is too abstract when thinking about the everyday lives of individuals who have experienced conflict and violence—and toward everyday social‐ecological connectivities. Sentient ecology offers a more “grounded” perspective on what resilience looks like—and on how sentience vis‐à‐vis the ecological can translate into everyday social expressions of resilience. Fundamentally, if resilience “has the potential to become a bridging concept between the natural and the social sciences and stimulate interdisciplinary dialogues and collaborations” (Davoudi, 2012, p. 306), sentient ecology is itself a potential bridging concept between the social and ecological.

Thinking about resilience within a sentient ecology frame reinforces the argument made at the outset that resilience is not about putting the onus on individuals to deal with whatever life throws at them and diminishing the responsibilities of governments and states. It is, rather, about the multiple connectivities between individuals and their social ecologies, which means recognizing “the bidirectional nature of influence in living systems” (Masten, 2001, p. 230) and, by extension, “the diverse capacities of matter (non‐human and [post]human) to affect and be affected” (Fox & Alldred, 2020, p. 280). It is submitted, therefore, that what sentient ecology more broadly highlights is the importance of developing resilience research—and in particular research on SES—in new posthumanist directions. Significantly, resilience scholars to date have given very little attention to posthumanism; and those who have engaged with the concept have been largely critical of it (see, for example, Chandler, 2014; Wakefield et al., 2021).

While acknowledging that posthumanism spans a very broad spectrum (Braidotti, 2018, p. 206), this article embraces the argument that “human beings are one of many components that make up our world, and that they cannot be understood apart from the wider relational assemblages, and the specific historical processes, of which they are part” (Crellin & Harris, 2021, p. 473). Part of Chandler’s (2014, p. 198) critique of posthumanism is that these assemblages themselves “are held to have real agency, rather than knowledgeable human subjects”, but this arguably creates a false dichotomy. Barad’s (2003, p. 817) work on posthumanism and new materialism underlines “agential intra‐actions” through which phenomena are produced, and the crucial point is that SES themselves can be conceptualized as complex relational assemblages that reflect agential intra‐actions (of which sentient ecology is one example). This, in turn, illuminates the scope for new interdisciplinary research on resilience that explores these intra‐actions, including between socio‐psychological and social‐ecological dynamics. As Barad (2003, p. 817) notes, “Boundaries do not sit still”.

In short, posthumanist ways of thinking about resilience can potentially make a significant contribution to the field. Cudworth and Hobden’s (2013, p. 449) argument that “A posthuman international relations will be attuned to the possibilities of a fuller range of actors and constraints in any given context” is no less pertinent to the study of resilience. Specifically with regard to CRSV, what this article has ultimately demonstrated through its discussion of sentient ecology is that posthumanism means, inter alia, thinking about “the aliveness, agency and co‐participation of ‘things’” (Tickner & Querejazu, 2021, p. 397) in the experiences of victims−/survivors and their neglected stories of resilience.

## CONFLICT OF INTEREST

No conflict of interest declared.

## ETHICS APPROVAL STATEMENT

This research received full ethics approval from the Humanities and Social Sciences Ethical Review Committee at the University of Birmingham, the European Research Council Executive Agency (ERCEA), the Ethics Committee at the Sarajevo School of Science and Technology, the Ethics Committee at Rosario University in Colombia, St. Mary’s Hospital Lacor in Uganda and the Ugandan National Council of Science and Technology (UNCST).

## Data Availability

The data are not publicly available but are deposited at https://doi.org/10.25500/edata.bham.00000705

## Endnotes

^1^
This article uses the terminology of victims−/survivors, reflecting the fact that some of the participants in this research identified more with the term “victims” while others primarily identified with the term “survivors”. Some of them, moreover, identified with both.

^2^
As a brief terminological note, Western (1982, p. 183) distinguishes between the environment and the ecological processes that take place within it. This article uses the term social ecologies to refer to “formal and informal social networks” (Ungar, 2013, p. 256), including families, communities, NGOs and land. These social ecologies, in turn, exist within and form part of a wider environment.

^3^
Other studies have examined resilience in the context of sexual violence in general, but not specifically sexual violence in conflict (see, for example, Catabay et al., 2019; Kaiser & Sinanan, 2020; Moletsane & Theron, 2017).

^4^
Zraly et al. (2013, p. 415), for example, define maternal resilience as “the capacity to overcome adverse mothering experiences”. Their primarily psychological approach to resilience is further reflected in their argument that “The Rwandan women in the study were stunningly resilient and creative in the wake of unfathomable violence” (Zraly et al., 2013, p. 435). Koos’ approach to resilience, in the context of his work on Sierra Leone, is largely socio‐psychological. Giving very little attention to the wealth of extant scholarship on resilience, he talks about “the resilience mechanism” (Koos, 2018, p. 201), which he does not fully define, and links this to his hypothesis that “The exposure to CRSV potentially inflicts stigma and shame on CRSV victims and their families. To reduce stigma and avert social exclusion, they will use countermeasures that manifest in higher levels of prosocial behavior” (Koos, 2018, p. 204).

^5^
The author (who is Principal Investigator), two postdoctoral researchers, several in‐country organizations and two independent psychologists in BiH and Colombia respectively were involved in applying the questionnaires.

^6^
Ruptures can, in some cases, cause significant turbulence within SES. Discussing forced cultural transformations, for example, Crane (2010) argues that “such localized cultural ruptures have the potential to spread upward through ‘revolution,’ upturning social–ecological systems more broadly”.

^7^
“During a back loop”, Holling (2004) argued, “unexpected interactions can occur among previously separate properties that can then nucleate an inherently novel and unexpected focus for future good or ill in the next cycle”.

^8^
No actual names are used in this research.
